# Chromatin Accessibility-Guided Targeting Identifies Structurally Constrained Regions in HBV cccDNA and Suppresses Viral Replication

**DOI:** 10.3390/ijms27156980

**Published:** 2026-08-03

**Authors:** Lianghao Kong, Sadahiro Iwabuchi, Ying-Yi Li, Kazuhisa Murai, Rie Korai, Kazunori Kawaguchi, Kouki Nio, Tadashi Imafuku, Tetsuro Shimakami, Taro Yamashita, Atsushi Tajima, Yutaka Suzuki, Masao Honda, Shinichi Hashimoto

**Affiliations:** 1 Department of Clinical Laboratory Medicine, Kanazawa University Graduate School of Medical Sciences, Kanazawa-shi 920-0942, Ishikawa, Japan; konglianghao@outlook.com (L.K.);; 2 Department of Bioinformatics and Genomics, Graduate School of Medical Sciences, Kanazawa University, Kanazawa-shi 920-8640, Ishikawa, Japan; iwabuchi@staff.kanazawa-u.ac.jp (S.I.);; 3 Department of Molecular Pathophysiology, Institute of Advanced Medicine, Wakayama Medical University, Wakayama-shi 641-8509, Wakayama, Japan; 4 Department of Gastroenterology, Graduate School of Medical Science, Kanazawa University, Kanazawa-shi 920-8640, Ishikawa, Japan; 5 Department of Computational Biology and Medical Sciences, Graduate School of Frontier Science, The University of Tokyo, Kashiwa-shi 277-8561, Chiba, Japan

**Keywords:** hepatitis B virus, cccDNA, chromatin accessibility, CRISPR/Cas9

## Abstract

Covalently closed circular DNA (cccDNA) is a stable episomal form of the hepatitis B virus (HBV) genome that serves as the template for viral transcription and replication and represents a major barrier to HBV cure. Here, we investigated chromatin accessibility patterns of cccDNA in HBV-infected hepatocyte cells at single-molecule resolution. We found that most cccDNA copies exhibited limited accessibility around nucleotides 800–1000, a region overlapping the polymerase open reading frame and the pregenomic RNA transcriptional region. Notably, a small subset of cccDNA showed detectable accessibility at this site, suggesting the presence of heterogeneous chromatin states. Based on this observation, we targeted this accessibility-associated region using a CRISPR/Cas9-based approach and observed reductions in HBV DNA-related signals, including cccDNA-enriched fractions and total HBV DNA levels across complementary experimental systems. These findings suggest that chromatin accessibility profiling may provide an additional framework for identifying candidate cccDNA target regions. Our study provides a proof-of-concept for accessibility-informed HBV targeting and supports further investigation of chromatin-associated vulnerability within HBV cccDNA.

## 1. Introduction

Hepatitis B virus (HBV) is a member of the Hepadnaviridae family and carries a relaxed circular DNA (rcDNA) genome of approximately 3200 base pairs. Following infection, rcDNA is transported to the nucleus, where it is repaired into covalently closed circular DNA (cccDNA), which exists as an episomal minichromosome associated with host histones and regulatory factors [1]. This cccDNA serves as the template for viral transcription and is essential for producing pregenomic RNA (pgRNA), ultimately leading to rcDNA synthesis and viral progeny generation [2,3]. Because cccDNA is highly stable and can persist for extended periods, with an estimated half-life exceeding nine months in HBV-infected patients, it represents a major barrier to achieving a complete cure for chronic HBV infection [4].

HBV cccDNA has been reported to form a chromatinized minichromosome associated with host histones and epigenetic modifications [3,5]. Nucleosome positioning and histone modifications on cccDNA have been reported to regulate viral transcriptional activity [6,7,8]. In addition, chromatin accessibility analyses have suggested that structural organization of cccDNA may influence transcriptional competence [6,8,9,10]. However, the structural organization of cccDNA at high resolution remains poorly understood. It remains unclear whether cccDNA molecules are structurally uniform or whether distinct chromatin states exist that influence transcriptional activity and susceptibility to genome editing. Addressing this question is essential for identifying candidate accessibility-associated regions that may serve as therapeutic targets.

Based on this concept, genome editing approaches targeting cccDNA have attracted considerable interest. Current antiviral therapies effectively suppress viral replication but rarely eliminate cccDNA. Therefore, strategies aimed at directly targeting cccDNA have attracted considerable interest. Genome editing approaches using CRISPR/Cas9 have been widely investigated as a potential strategy to disrupt cccDNA and reduce viral persistence [11,12,13,14,15,16,17,18,19,20]. Previous studies have proposed several targetable regions within the HBV genome, including the overlapping polymerase and surface open reading frame (ORF) region, the enhancer II and X protein region, and the overlapping polymerase and core ORF region [14]. Targeting these regions with guide RNAs (gRNAs) has been shown to reduce both cccDNA and HBV DNA levels, with dual gRNA strategies generally demonstrating improved efficacy compared to single gRNAs [12,18,19]. However, most studies have selected gRNA target sites based primarily on sequence conservation or functional annotation, without considering the structural organization or chromatin accessibility of cccDNA.

In this study, we investigated chromatin accessibility patterns of HBV cccDNA at single-molecule resolution in HBV-infected hepatocytes. We identified an accessibility-associated region around nucleotides 800–1000 that exhibited relatively reduced chromatin accessibility in most cccDNA molecules, while a small subset displayed detectable accessibility, suggesting heterogeneous chromatin states. Targeting this accessibility-associated region using CRISPR/Cas9 resulted in reductions in HBV DNA-related signals across complementary experimental systems. These findings suggest that chromatin accessibility profiling may provide an additional framework for identifying candidate cccDNA target regions.

## 2. Results

### 2.1. Rarely Open Chromatin Regions in HBV cccDNA

**Figure 1:**
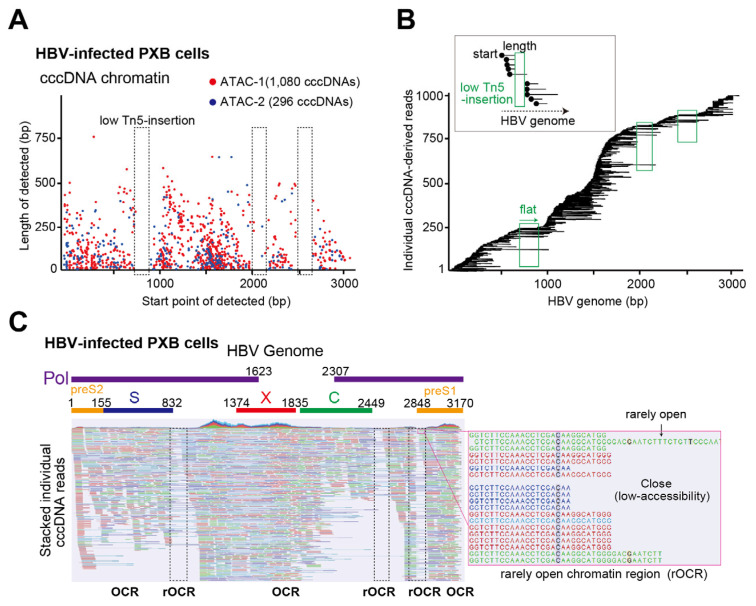
Identification of rarely open chromatin regions (rOCRs) in hepatitis B virus (HBV) covalently closed circular DNA (cccDNA). (**A**) Distribution of assay for transposase-accessible chromatin using sequencing (ATAC-seq)-derived Tn5 insertion sites mapped to the HBV genome. Each dot represents an individual cccDNA-derived ATAC-seq fragment plotted according to its genomic start position and fragment length. Three genomic intervals (approximately 800–1000 bp, 2000–2200 bp, and 2500–2700 bp) showed markedly reduced Tn5 insertion frequencies, suggesting candidate regions with locally reduced chromatin accessibility. (**B**) Visualization of individual cccDNA-derived reads projected onto the HBV genome. Each horizontal line represents one ATAC-seq fragment extending from its insertion site to its fragment end. This intermediate representation links the genome-wide distribution shown in panel (**A**) with the stacked-read visualization shown in panel (**C**). Regions where read coverage becomes locally flat correspond to candidate low-accessibility regions. (**C**) Stacked visualization of individual cccDNA-derived reads across the HBV genome. Each horizontal line represents one cccDNA-derived ATAC-seq fragment. Regions exhibiting consistently reduced read coverage were operationally defined as rarely open chromatin regions (rOCRs). Only a limited subset of reads extended across these regions, whereas most fragments terminated before entering them, indicating locally reduced chromatin accessibility.

Assay for transposase-accessible chromatin using sequencing (ATAC-seq) analysis of HBV-infected PXB cells revealed discrete regions of reduced chromatin accessibility along the HBV genome. Because the HBV used for infection of PXB cells belonged to genotype C2, a patient-derived HBV reference genome of the same lineage (PV210398) was used for the initial mapping. Mapping of Tn5 insertion start sites identified three genomic intervals spanning approximately nucleotides 800–1000, 2000–2200, and 2500–2700, where read initiation was markedly reduced (Figure 1A). These regions exhibited limited Tn5 insertion events, suggesting locally reduced chromatin accessibility within HBV cccDNA. To better visualize how individual sequencing fragments contributed to this distribution, the start position and fragment length of each ATAC-seq fragment were projected onto the HBV genome (Figure 1B). This intermediate representation revealed regions where read coverage became locally flat, corresponding to candidate low-accessibility regions. Stacked visualization of individual cccDNA-derived reads further demonstrated that these regions were inaccessible in the majority of cccDNA molecules (Figure 1C). Only a limited subset of reads extended across these regions, whereas most fragments terminated before entering them. Based on these observations, these regions were operationally defined as rarely open chromatin regions (rOCRs), representing regions of relatively reduced chromatin accessibility. Although similar accessibility patterns were suggested in our previous study [21], the present visualization provides a clearer definition of rOCRs at the level of individual cccDNA molecules.

**Figure 2:**
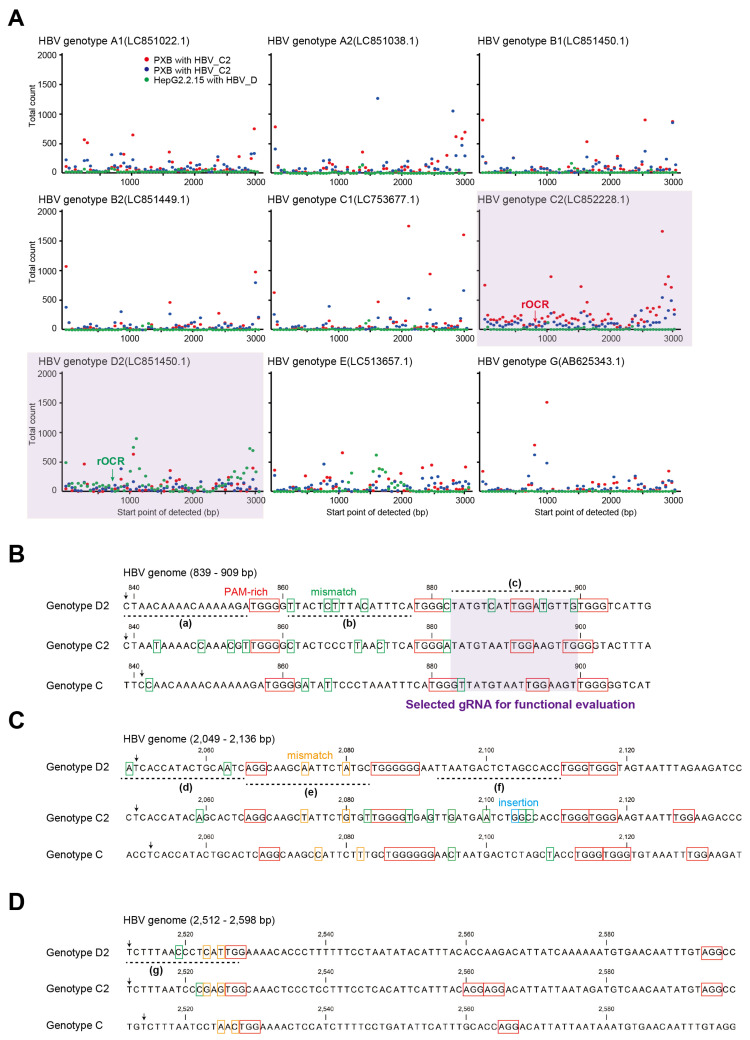
Guide RNA selection based on chromatin accessibility and sequence conservation across representative HBV genotypes. (**A**) Distribution of ATAC-seq-derived reads mapped to representative HBV genotypes and experimental systems. The candidate target region identified in the HBV genotype C2 infection model is highlighted (purple shading) and compared with the corresponding genomic regions in other HBV genotypes. The corresponding region in genotype D2 is also highlighted because HepG2.2.15 cells harbor genotype D-derived HBV DNA and were used for subsequent functional analyses. (**B**–**D**) Sequence comparison of the three candidate rOCRs identified in panel (**A**). The purple-shaded region corresponds to the highlighted candidate target region shown in panel (**A**), providing a visual link between chromatin accessibility profiling and guide RNA selection. Conserved protospacer adjacent motif (PAM)-rich motifs, sequence mismatches, and insertions among HBV genotypes are indicated. Based on sequence conservation and PAM distribution across the experimental systems used in this study, the guide RNA (gRNA) targeting the 839–909 bp region (panel (**B**)) was selected for functional evaluation. Purple shading indicates the accessibility-associated region highlighted in panel A and the corresponding sequence region shown in panels (**B**–**D**), providing a visual link between chromatin accessibility profiling and gRNA selection. Lowercase labels (a–g) indicate representative candidate guide RNA target sites evaluated within these regions. Among these candidates, the site labeled (c) in panel B (839–909 bp) was selected for subsequent functional analyses.

To investigate whether these accessibility-associated regions were conserved among different HBV genotypes and suitable for guide RNA design, the corresponding genomic regions were subsequently compared across representative HBV genotypes and experimental systems (Figure 2).

### 2.2. Functional Perturbation of Rarely Open Chromatin Regions by CRISPR/Cas9

To investigate whether the accessibility-associated regions identified in HBV-infected PXB cells could serve as candidate targets for CRISPR/Cas9-mediated perturbation, we next designed guide RNAs based on the three rOCRs identified by single-cell ATAC-seq. Because the HBV used for infection of PXB cells belonged to genotype C2, the corresponding genomic regions were compared across representative HBV genotypes (D2, C2, and C) to evaluate sequence conservation and guide RNA compatibility (Figure 2). Figure 2A summarizes the distribution of ATAC-seq-derived reads mapped to representative HBV genotypes and experimental systems. The accessibility-associated region identified in the genotype C2 infection model is highlighted and compared with the corresponding genomic regions in the other HBV genotypes. The corresponding region in genotype D2 is also highlighted because genotype D-derived HBV sequences were used in complementary experiments described below. Figure 2B–D show sequence alignments of the three candidate rOCRs identified in Figure 2A. In the 839–909 bp region (Figure 2B), multiple protospacer adjacent motif (PAM)-rich motifs were conserved across genotypes despite only limited nucleotide mismatches. Because CRISPR/Cas9 generally tolerates a small number of mismatches outside the seed region, this region was considered suitable for guide RNA (gRNA) design. In contrast, the 2049–2136 bp region (Figure 2C) exhibited greater sequence variation, including a genotype C2-specific insertion, whereas the 2512–2598 bp region (Figure 2D) contained fewer conserved PAM motifs, making these regions less favorable for broadly conserved guide RNA targeting.

Based on these observations, multiple candidate guide RNA sequences were further evaluated by BLASTN (NCBI; accessed in July 2026) analysis (Supplementary Table S1), in which representative hits are shown and HBV-related sequences are highlighted in blue. Among the candidate regions, guide RNAs derived from the 839–909 bp region showed the highest HBV specificity together with favorable PAM distribution and sequence conservation. Accordingly, a guide RNA targeting the PAM-rich region spanning approximately 833–899 bp was selected for subsequent functional analyses. Because primary PXB cells cannot be serially passaged and are not suitable for stable Cas9 introduction, functional validation of the selected guide RNA was first performed using the NTCP-overexpressing Huh7 (Huh7-NTCP) cell de novo infection model infected with the same HBV genotype C2 strain used for the PXB experiments. HepG2.2.15 cells, which support persistent HBV replication, were subsequently used as a complementary model for repeated molecular and genomic analyses.

### 2.3. CRISPR/Cas9 Targeting of an Accessibility cccDNA Region Suppresses HBV Replication in the De Novo Infection Model

**Figure 3:**
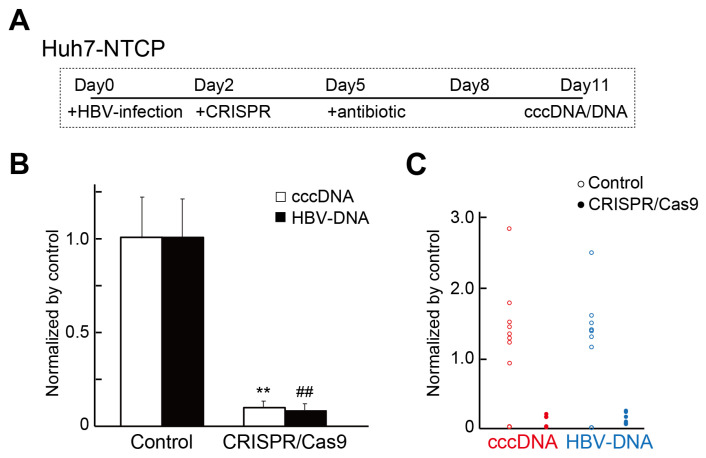
Functional validation of the selected accessibility-associated guide RNA in the HBV de novo infection model. (**A**) Experimental design for CRISPR/Cas9-mediated targeting in HBV-infected Huh7-NTCP cells. Following identification of the accessibility-associated target region in HBV-infected PXB cells (Figure 1 and Figure 2), the selected guide RNA was evaluated in Huh7-NTCP cells infected with the same HBV genotype C2 strain. Cells were infected with HBV on Day 0, exposed to the target-specific gRNA lentivirus on Day 2, cultured under antibiotic selection from Day 5, and harvested on Day 11 for quantification of cccDNA and total HBV DNA. (**B**) Quantitative PCR analysis of cccDNA (open bars) and total HBV DNA (filled bars) in Huh7-NTCP cells. Values were normalized to the corresponding control group. CRISPR/Cas9 targeting of the accessibility-associated region significantly reduced both cccDNA and total HBV DNA. Data are presented as mean ± standard error (SE). ** indicates *p* < 0.01 for cccDNA-associated fractions compared with the control group, whereas ## indicates *p* < 0.01 for total HBV DNA compared with the control group. (**C**) Individual data points corresponding to the quantitative PCR results shown in panel (**B**). Open circles represent control samples and filled circles represent CRISPR/Cas9-treated samples, demonstrating reproducible reductions in both cccDNA and total HBV DNA across independent experiments.

Finally, to determine whether the selected accessibility-associated target region could be functionally perturbed during de novo HBV infection, Huh7-NTCP cells were infected with the same HBV genotype C2 inoculum used in the PXB experiments and subsequently subjected to CRISPR/Cas9 targeting (Figure 3A). CRISPR/Cas9 targeting resulted in significant reductions in both cccDNA and total HBV DNA compared with control cells (Figure 3B). Individual data points from independent experiments showed reproducible decreases in both viral DNA species (Figure 3C). These findings demonstrate that CRISPR/Cas9 targeting of the accessibility-associated region suppresses HBV replication in a de novo HBV infection model that closely corresponds to the system used for rOCR identification.

### 2.4. Complementary Molecular Analyses in HepG2.2.15 Cells

**Figure 4:**
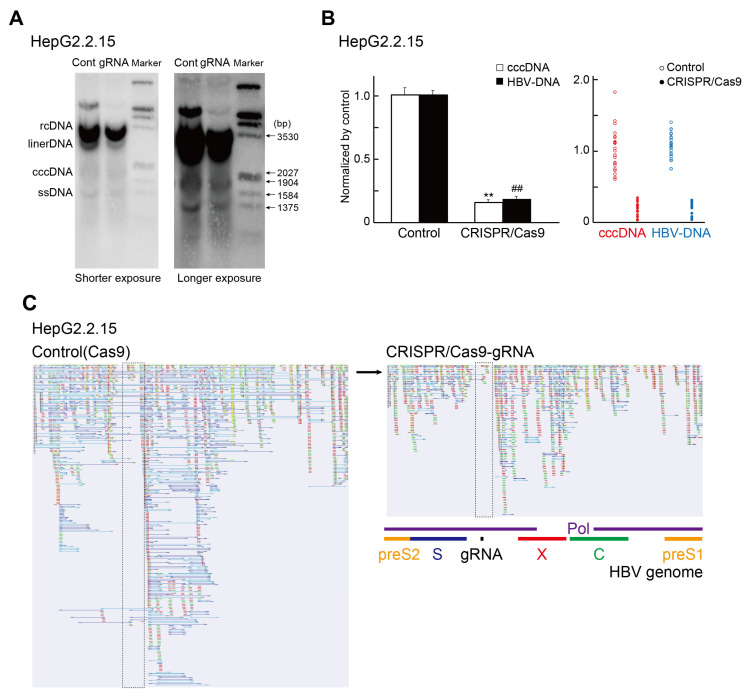
Complementary molecular analyses of CRISPR/Cas9 targeting in HepG2.2.15 cells. (**A**) Southern blot analysis of HBV DNA species in HepG2.2.15 cells following CRISPR/Cas9 targeting. Relaxed circular DNA (rcDNA), linear DNA, cccDNA, and single-stranded DNA (ssDNA) are indicated. Both short- and long-exposure images are shown. (**B**) Quantification of cccDNA-associated fractions and total HBV DNA levels in HepG2.2.15 cells by qPCR. Bar graphs show values normalized to the control group. ** Data are presented as mean ± SE. *p* < 0.01 for cccDNA-associated fractions and ## *p* < 0.01 for total HBV DNA compared with the control group. Individual measurements are overlaid as dot plots, with red indicating cccDNA-associated fractions and blue indicating total HBV DNA. Open circles represent control cells (Cas9 only), whereas filled circles represent CRISPR/Cas9-treated cells. (**C**) Bulk ATAC-seq reads mapped to the HBV genome in HepG2.2.15 cells. Stacked read visualization shows reduced ATAC-seq read coverage around the CRISPR/Cas9 target region following treatment, consistent with perturbation of the corresponding HBV genomic locus. Control (**left panel**) and CRISPR/Cas9-treated (**right panel**). The HBV genome annotation displayed below the right panel applies to both the control and CRISPR/Cas9-treated samples.

Because Huh7-NTCP cells represent a de novo HBV infection model primarily suited for evaluating antiviral activity during early infection, complementary molecular analyses were subsequently performed in HepG2.2.15 cells, which provide a stable experimental system for repeated mechanistic characterization. Southern blot analysis detected multiple HBV DNA species, including relaxed circular DNA (rcDNA), linear DNA, cccDNA, and single-stranded DNA (ssDNA). CRISPR/Cas9 targeting of the selected accessibility-associated region resulted in a modest reduction in cccDNA-associated signals together with decreases in other HBV DNA species compared with control cells (Figure 4A). To quantitatively evaluate these effects, cccDNA and total HBV DNA were measured by quantitative PCR. Consistent with the observations in Huh7-NTCP cells (Figure 3), CRISPR/Cas9 targeting significantly reduced both cccDNA and total HBV DNA in HepG2.2.15 cells (Figure 4B). These findings indicate that the antiviral activity observed in the de novo infection model can also be reproduced in a stable HBV replication model. To examine chromatin changes associated with CRISPR/Cas9 treatment, bulk ATAC-seq reads were visualized as stacked fragments across the HBV genome. Following CRISPR/Cas9 targeting, reads spanning the selected accessibility-associated region were markedly reduced, indicating disruption of the previously identified rOCR configuration (Figure 4C). These chromatin-level alterations were consistent with the reduction in viral DNA observed by Southern blotting and quantitative PCR. To further evaluate whether the observed antiviral effects were specific to the selected target region, an additional gRNA targeting the 1200-bp region was designed based on the bulk ATAC-seq profile (Supplementary Figure S1). Comparison of Southern blot signals demonstrated that the original gRNA-800 produced a greater reduction in cccDNA-associated signals than gRNA-1200 (Supplementary Figure S2). Although this comparison does not establish a causal role for chromatin accessibility, it supports the possibility that antiviral efficacy differs according to the targeted HBV genomic region. Because the selected CRISPR/Cas9 target overlaps the pregenomic RNA (pgRNA) transcription unit, we next examined whether RNA interference directed against the same region produced comparable effects. Based on our previous systematic small interfering RNA (siRNA) screening of the HBV genome [21], an siRNA targeting nucleotide 859 was selected. Although the siRNA alone reduced pgRNA expression, combining the siRNA with CRISPR/Cas9 targeting did not produce an additional reduction (Supplementary Figure S3), suggesting that the antiviral effects of CRISPR/Cas9 cannot be explained solely by transcriptional suppression. Finally, to determine whether CRISPR/Cas9 activity was distinct from inhibition of reverse transcription, cells were treated with entecavir. While entecavir markedly reduced HBV DNA levels, CRISPR/Cas9 treatment produced an additional decrease in HBV DNA and showed a trend toward further reduction in cccDNA (Supplementary Figure S4). These findings suggest that CRISPR/Cas9-mediated targeting acts through a mechanism distinct from reverse transcription inhibition and may further reduce residual viral DNA remaining after nucleoside analogue treatment.

### 2.5. Host Chromatin and Transcriptional Responses Following CRISPR/Cas9 Targeting

**Figure 5:**
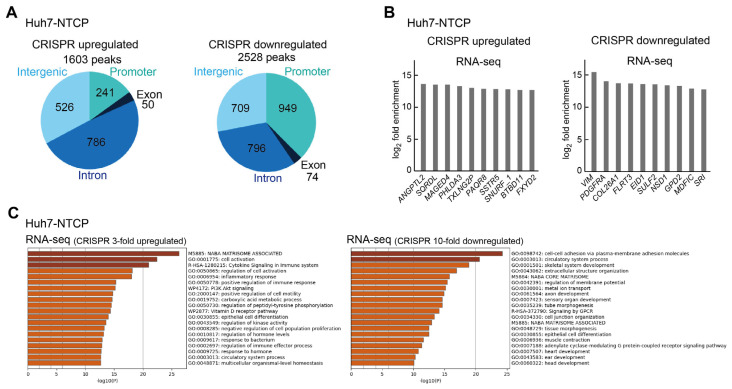
Genome-wide host chromatin and transcriptional responses following CRISPR/Cas9 targeting in HBV-infected Huh7-NTCP cells. (**A**) Genome-wide annotation of differentially accessible chromatin regions identified by ATAC-seq following CRISPR/Cas9 treatment. Pie charts show the genomic distribution of regions exhibiting increased (**left**) or decreased (**right**) chromatin accessibility relative to control cells. (**B**) Top host genes exhibiting increased (**left**) or decreased (**right**) expression following CRISPR/Cas9 treatment, identified by RNA-seq analysis. Genes are ranked according to log_2_ fold change. (**C**) Metascape enrichment analysis of differentially expressed genes. The left panel shows pathways enriched among genes upregulated more than threefold following CRISPR/Cas9 treatment, whereas the right panel shows pathways enriched among genes downregulated more than tenfold. Functional categories associated with extracellular matrix organization, cytokine signaling, immune responses, cell adhesion, and developmental processes were significantly enriched.

Following validation of antiviral activity, we next investigated whether CRISPR/Cas9 targeting of the accessibility-associated HBV region was accompanied by broader changes in host chromatin accessibility and gene expression. Genome-wide ATAC-seq and RNA-sequencing (RNA-seq) analyses were performed in Huh7-NTCP cells following CRISPR/Cas9 treatment. Genome-wide ATAC-seq identified numerous regions exhibiting altered chromatin accessibility following CRISPR/Cas9 treatment (Figure 5A). Differential RNA-seq analysis further revealed host genes that were either upregulated or downregulated in response to CRISPR/Cas9-mediated targeting (Figure 5B). Functional enrichment analysis of differentially expressed genes demonstrated significant enrichment of pathways associated with cell morphogenesis, cytoskeletal organization, cell junction assembly, and RHO GTPase signaling (Figure 5C). These findings indicate that CRISPR/Cas9-mediated perturbation of the accessibility-associated HBV region is accompanied by coordinated remodeling of host chromatin architecture and transcriptional programs. Representative changes in chromatin accessibility are illustrated at the *NAP1L1* locus (Supplementary Figure S5). Comparison of ATAC-seq and RNA-seq datasets identified several genes exhibiting concordant chromatin accessibility and transcriptional changes following CRISPR/Cas9 treatment (Supplementary Figure S6). Metascape analysis further demonstrated enrichment of pathways related to cellular morphogenesis, signaling, and cytoskeletal organization among differentially accessible chromatin regions (Supplementary Figure S7). To determine whether similar host responses were observed in an independent HBV experimental system, complementary RNA-seq and ATAC-seq analyses were performed in HepG2.2.15 cells (Supplementary Figure S8). Although the specific genes differed between the two models, coordinated alterations in host chromatin accessibility and transcriptional profiles were observed following CRISPR/Cas9 treatment in both experimental systems.

## 3. Discussion

Single-cell chromatin accessibility profiling has suggested that HBV cccDNA is not structurally uniform but instead contains discrete regions that remain relatively inaccessible across individual molecules during early infection [21]. While previous studies have demonstrated nucleosome positioning and epigenetic regulation of the HBV minichromosome, chromatin accessibility at the single-molecule level has remained poorly characterized [3,5,6,9]. More recent chromatin mapping studies using MNase-seq and ATAC-seq further indicate that HBV cccDNA adopts heterogeneous nucleosome configurations and dynamic chromatin states associated with transcriptional activity [8,22]. In addition, only a fraction of cccDNA copies appears to adopt relatively accessible chromatin configurations, whereas the majority remain in less accessible states. This heterogeneity may influence susceptibility to genome editing and transcriptional regulation [23]. Together, these studies support the concept that chromatin accessibility represents an additional layer of cccDNA regulation.

In the present study, ATAC-seq analysis identified three reproducible regions of reduced chromatin accessibility within the HBV genome that consistently exhibited relatively low accessibility across multiple experimental systems. These findings further support previous observations that HBV cccDNA exists in heterogeneous chromatin states rather than as a uniformly accessible template. While prior CRISPR/Cas9 studies have primarily selected target sites according to sequence conservation or coding function, exemplified by gRNA-5 targeting nucleotides 1179–1197 bp [16], the rOCR identified here was selected using single-cell chromatin accessibility profiling, thereby incorporating chromatin accessibility as an additional criterion for guide RNA selection. Unlike conventional CRISPR strategies targeting conserved ORF regions such as S/Pol, X/Pol, or Core [14,16,18], our approach evaluated whether chromatin accessibility profiling could provide complementary information for identifying candidate target sites. However, the present study does not distinguish whether the observed antiviral effects resulted from accessibility-associated vulnerability, disruption of functionally important HBV sequences, or a combination of both mechanisms. Accordingly, the present findings should be interpreted as a proof-of-concept demonstration that chromatin accessibility profiling may serve as a complementary framework for identifying candidate cccDNA target regions. Future studies using accessibility-independent control regions, systematic comparisons among multiple guide RNAs, and reconstructed cccDNA chromatin systems will be important for clarifying the relationship between chromatin organization and susceptibility to genome editing.

Previous studies have shown that CRISPR-mediated editing of HBV coding regions can suppress viral replication by generating defective pgRNA transcripts, although the antiviral effects reported to date have generally been partial [19]. In the present study, targeting of an accessibility-associated region was likewise associated with reductions in cccDNA-associated fractions and total HBV DNA levels across complementary HBV experimental systems, including both persistent replication and de novo infection models. However, the present study does not distinguish whether these effects resulted from preferential targeting of transcriptionally competent cccDNA, disruption of functionally important viral genomic elements, or a combination of both mechanisms. Consequently, our findings should be interpreted as supporting the possibility that chromatin accessibility profiling may provide complementary information for identifying candidate HBV target regions, rather than demonstrating that chromatin accessibility alone determines CRISPR/Cas9 efficacy. Consistent with this interpretation, combined treatment with the nucleoside analogue entecavir further reduced HBV DNA-related signals, including cccDNA-associated fractions. In addition, siRNA targeting of the same genomic region produced partially overlapping effects on pgRNA expression. Together, these complementary observations are consistent with the possibility that CRISPR/Cas9-mediated perturbation of accessibility-associated regions influences HBV maintenance through mechanisms that are not fully explained by reverse transcription inhibition or transcriptional suppression alone. Nevertheless, further validation using additional guide RNAs, primary hepatocyte models, and accessibility-independent control regions will be required to clarify the underlying mechanisms.

To further characterize the host cellular response following cccDNA targeting, we performed bulk ATAC-seq and RNA-seq analyses. CRISPR-mediated disruption of the rOCR-associated region (839–909 bp) was associated with broad changes in host chromatin accessibility, whereas transcriptional changes were comparatively more limited and were primarily enriched in immune- and cytokine-related pathways. This dissociation between chromatin accessibility and transcriptional output may reflect temporal delays, indirect responses to viral suppression, or post-transcriptional regulatory mechanisms. Among the affected loci, *NAP1L1* and *CEP68* exhibited altered chromatin accessibility and/or gene expression, suggesting potential involvement in host chromatin-associated responses following cccDNA perturbation. NAP1L1 has not previously been implicated in HBV or cccDNA regulation. However, as a nucleosome assembly protein and histone chaperone, *NAP1L1* may influence the chromatin organization of episomal DNA, raising the possibility that it contributes to cccDNA-associated chromatin dynamics [24,25,26]. In our previous study, CEP290 was identified as a regulator of chromatin accessibility at the same rOCR-associated region [21]. Therefore, the identification of an additional CEP family-related gene in the present analysis is noteworthy. Although the functional relationship between CEP family proteins and cccDNA chromatin regulation remains unclear, these findings raise the possibility that nuclear structural or chromatin-associated factors contribute to the maintenance of heterogeneous cccDNA chromatin states. Gene ontology analyses further highlighted distinct biological responses at the chromatin and transcriptional levels. Changes in chromatin accessibility were enriched in pathways related to nuclear organization, cytoskeletal architecture, and cellular morphology, whereas transcriptomic alterations were more strongly associated with immune- and cytokine-related pathways. These findings suggest that cccDNA perturbation is accompanied by multilayered host cellular responses involving both chromatin-associated remodeling and transcriptional immune activation. Collectively, these observations raise the possibility that accessibility-associated cccDNA targeting influences host chromatin states beyond direct perturbation of the viral genome alone.

Several limitations should be considered. First, HepG2.2.15 cells contain integrated HBV DNA, which may contribute to HBV DNA-derived signals detected in the present study. In addition, qPCR-based quantification following nuclease treatment cannot completely exclude contributions from non-cccDNA HBV DNA species. Therefore, the measured signals should be interpreted as cccDNA-associated fractions rather than definitive quantification of pure episomal cccDNA alone. However, similar trends were also observed in complementary de novo infection systems, including Huh7-NTCP cells infected with the same HBV inoculum used for PXB cells, suggesting that perturbation of accessibility-associated regions may influence HBV maintenance across multiple experimental contexts. Second, the present study does not fully distinguish whether the observed antiviral effects were specifically associated with chromatin accessibility or instead reflected disruption of functionally important HBV genomic regions, including the overlapping polymerase/pgRNA region. Because the targeted 800–1000 bp interval contains functionally important viral elements, both mechanisms may have contributed to the observed effects. Future studies comparing accessibility-associated regions with accessibility-independent control regions and additional guide RNAs will be important for clarifying this relationship. Third, comprehensive genome-wide off-target analyses, such as GUIDE-seq or Digenome-seq, were not performed in the current study. Although bulk ATAC-seq and RNA-seq analyses revealed biologically coherent changes in chromatin accessibility and gene expression, these observations cannot exclude potential off-target effects. Dedicated genome-wide off-target analyses and computational validation will therefore be necessary in future studies, particularly in the context of therapeutic development. Fourth, although an additional guide RNA targeting the 1200-bp region was evaluated, the present study did not systematically compare multiple independently selected guide RNAs across accessibility-associated and accessibility-independent regions. Future studies incorporating larger guide RNA panels and combinatorial targeting strategies will therefore be important for determining whether accessibility-associated targeting provides advantages beyond conventional sequence-based approaches. Fifth, the conservation of accessibility-associated regions across HBV genotypes remains incompletely understood. Although broadly similar reduced-accessibility patterns were observed across the examined reference genomes, the extent to which these accessibility-associated regions are functionally conserved remains unclear. Because cccDNA chromatin organization may differ depending on viral genotype, infection state, and host cellular context, further studies using genotype-matched infection systems and primary hepatocyte models will be important.

Despite these limitations, the present findings support the possibility that HBV cccDNA contains heterogeneous chromatin accessibility states that can be explored using single-molecule chromatin accessibility profiling. Furthermore, CRISPR/Cas9 perturbation of accessibility-associated regions was consistently associated with reductions in HBV DNA-related signals across complementary HBV experimental systems. Collectively, these findings support the use of chromatin accessibility profiling as a proof-of-concept framework for identifying candidate cccDNA target regions for future investigation.

## 4. Materials and Methods

### 4.1. Cell Culture and HBV Infection

Primary human hepatocytes (PXB cells), HBV inoculum, and culture medium were obtained from PhoenixBio Co., Ltd. (Hiroshima, Japan). PXB cells were incubated for 6 h and then infected with HBV at a multiplicity of infection (MOI) of 5 genome equivalents (GEq)/cell in the presence of 5% polyethylene glycol 8000 (Hampton Research, Aliso Viejo, CA, USA) for 1–2 days at 37 °C in a 5% CO_2_ humidified incubator. After removal of the supernatant, cells were maintained in fresh medium. Culture medium was replaced 4–5 days after the initial change. Cells were used for single-cell ATAC-seq 10–12 days post-infection. Under these relatively early infection conditions, HBV-derived nuclear signals were expected to predominantly reflect episomal HBV DNA species rather than long-term integrated HBV DNA. The human liver cancer cell line HepG2.2.15 (authenticated by DNA fingerprinting in 2016) was cultured on collagen I-coated plates (AGC TECHNO GLASS Co., Ltd., Shizuoka, Japan) in DMEM/F-12 with GlutaMAX supplement (Thermo Fisher Scientific, Waltham, MA, USA), supplemented with 10% fetal bovine serum (Hyclone Laboratories, Logan, UT, USA), 100 U/mL penicillin, 100 μg/mL streptomycin, and 400 μg/mL G418 (Thermo Fisher). NTCP-overexpressing Huh7 cells (Huh7-NTCP) were cultured in the same medium without G418 [10]. Because stable Cas9 introduction and repeated perturbation experiments were technically difficult in primary PXB cultures, complementary HBV experimental systems including HepG2.2.15 and Huh7-NTCP cells were used for subsequent functional analyses.

### 4.2. ATAC-Seq Library Preparation and Sequencing in PXB Cells

Nuclei were isolated from HBV-infected PXB cells using the Chromium Next GEM Single Cell ATAC Reagent Kits v1.1 (10x Genomics, Pleasanton, CA, USA). Briefly, approximately 1 × 10^6^ cells were scraped into a 1.5-mL microcentrifuge tube and centrifuged at 500× *g* for 5 min at 4 °C. The supernatant was removed, and 100 μL of cold lysis buffer was added. After 15 min on ice, 1 mL of chilled wash buffer was added. Nuclei were pelleted again and resuspended in 200 μL of cold Nuclei Buffer to a final concentration of about 3000 nuclei/μL. Single-cell ATAC-seq libraries were prepared following the Chromium user guide. Nuclei were partitioned into gel bead-in-emulsions (GEMs) using a Chromium Controller (Chip H). Indexed libraries were generated via PCR using Single Index Kit (Plate N, 10x Genomics) with the following cycling protocol: 98 °C for 45 s, 12 cycles of 98 °C for 20 s, 67 °C for 30 s, 72 °C for 20 s, and a final extension at 72 °C for 60 s. Libraries were purified using SPRIselect beads (Beckman Coulter, Brea, CA, USA) and sequenced on a NovaSeq 6000 using SP Reagent Kit v1.5 (100 cycles; Illumina, San Diego, CA, USA) with the following read structure: 50 bp (Read 1), 8 bp (i7 index), 24 bp (i5 index), and 50 bp (Read 2). Raw FASTQ files were generated using Cell Ranger ATAC v2.1.0. Detection of HBV reads was performed using CLC Genomics Workbench v25.0.1 (Qiagen, Hilden, Germany) with reference to HBV genomes shown in Supplementary Table S1. cccDNA was not physically isolated prior to scATAC-seq analysis. Instead, HBV-infected PXB cells were analyzed at 10–12 days post-infection, and cells harboring HBV genomic reads were identified computationally. Under these relatively early infection conditions, HBV-derived nuclear signals were expected to predominantly reflect episomal HBV DNA species, whereas long-term integrated HBV DNA was not expected to substantially contribute to the detected signals. Reduced ATAC-seq signal was interpreted as relatively reduced chromatin accessibility rather than definitive evidence of fully closed chromatin states.

### 4.3. The gRNA Design and Lentiviral Delivery

A custom gRNA expression construct was generated using the pRSG16Bla-U6-sg-UbiC-TagRep-2A-Blast plasmid backbone (Cellecta, Inc., Mountain View, CA, USA). The gRNA sequence targeting cccDNA was 5′-GTTATGTCATTGGATGTTAT-3′. Cas9 expression lentivirus (pRCCH-CMV-Cas9-2A-Hygro, Cat. #SVC9-PS) was transduced into HepG2.2.15 or Huh7-NTCP cells to generate stable Cas9-expressing lines. For gRNA delivery, 300 μL of gRNA lentiviral particles (2 × 10^6^ TU) and 2 μL hexadimethrine bromide (Sigma-Aldrich, St. Louis, MO, USA) were added to 1 mL of culture medium and incubated for 3 days. Media was then replaced, and cells were cultured further. In experiments assessing CRISPR effects on cccDNA post-infection, Huh7-NTCP cells were first infected with HBV for 2 days, after which the medium was replaced with gRNA lentivirus-containing medium. The targeted region within the approximately 800–1000 bp interval overlapped the HBV polymerase open reading frame and corresponded to a region showing relatively reduced Tn5 insertion frequency in the scATAC-seq analysis. The purpose of targeting this region in the present study was not to demonstrate superiority over previously reported polymerase-targeting strategies, but rather to evaluate whether chromatin accessibility profiling could identify accessibility-associated candidate loci within HBV cccDNA. The effect of CRISPR/Cas9 perturbation on HBV DNA-associated signals was evaluated by bulk ATAC-seq together with qPCR and Southren blot analyses. Specifically, reduced ATAC-seq reads mapping around the targeted region were interpreted as being consistent with perturbation of the corresponding HBV genomic locus. Because direct measurement of indel frequency within low-abundance episomal cccDNA remains technically challenging, CRISPR/Cas9-mediated effects were inferred indirectly using complementary molecular and chromatin accessibility analyses. To minimize potential effects associated with lentiviral exposure, all comparisons were performed within the same stable Cas9-expressing cell background, with delivery of target-specific gRNA representing the primary experimental variable. To assess whether the observed effects were specific to the accessibility-associated target region, an additional guide RNA targeting an approximately 1200-bp region, which exhibited comparatively higher ATAC-seq read coverage, was designed based on PAM availability, sequence conservation, and guide specificity. The corresponding gRNA was introduced into HepG2.2.15 cells using the same lentiviral procedure described above, and HBV DNA-associated signals were evaluated by Southern blot analysis under identical experimental conditions (Supplementary Figures S1 and S2).

### 4.4. Quantification of cccDNA and HBV DNA

For Southern blotting, Hirt-extracted HBV DNA fractions were prepared from HepG2.2.15 cells, Control (Cas9-transfected) cells and CRISPR/Cas9-treated cells using the Hirt protein-free DNA extraction method. Hirt-extracted DNA (40 µg) was subjected to Southern blot analysis to detect HBV DNA species, including cccDNA-associated signals. Digoxigenin-labeled single-stranded RNA probes generated with a DIG RNA Labeling kit (Roche Diagnostics GmbH, Mannheim, Germany) and hybridization conditions were prepared as previously reported [27]. The qPCR-based quantification of cccDNA-associated fractions and total HBV DNA was performed as described previously [10,21]. In brief, total DNA was extracted using SMITEST EX-R&D (Medical & Biological Laboratories, Tokyo, Japan) and quantified with a NanoDrop spectrophotometer (Thermo Fisher). The cccDNA-enriched fractions were prepared from 1200 ng DNA by Plasmid-Safe ATP-Dependent DNase (Lucigen, Middleton, MD, USA) treatment (37 °C, 45 min; 70 °C, 30 min) [28]. TaqMan qPCR with primers 5′-ACTCACCAACCTCCTGTCCT-3′ and 5′-GACAAACGGGCAACATACCT-3′ and probe 5′-/56-FAM/TATCGCTGG/ZEN/ATGTGTCTGCGGCGT/3IABkFQ/-3′ (Integrated DNA Technologies, Coralville, IA, USA) was performed (50 °C 2 min; 95 °C 2.5 min; 50 cycles of 95 °C 3 s, 60 °C 30 s). Copy numbers were derived from standard curves and normalized to input DNA to minimize batch effects. Plasmid-Safe DNase treatment was used to preferentially remove non-cccDNA species; however, qPCR-based quantification cannot completely discriminate among all HBV DNA intermediates [29,30]. Therefore, these measurement were interpreted as cccDNA-associated fractions and evaluated together with complementary Southern blot and chromatin accessibility analyses.

### 4.5. Bulk ATAC-Seq and RNA-Seq Analysis

Bulk ATAC-seq was performed using the ATAC-Seq Kit (Active Motif, Carlsbad, CA, USA). Briefly, 1 × 10^5^ cells were pelleted, lysed, and subjected to transposition. DNA was purified, amplified, and purified again using SPRIselect beads. Library quality was assessed using Agilent 4200 TapeStation and Qubit 2.0 (Thermo Fisher). Sequencing was performed on a NextSeq 500/550 (Illumina, 75 paired end). FASTQ files were uploaded to Active Motif’s Basepair portal and analyzed for peak comparison between control and CRISPR/gRNA-treated cells. Results are in Supplementary Data. Total RNA was extracted using the RNeasy Mini Kit (Qiagen). RNA quality and concentration were assessed with the Agilent TapeStation and Qubit. Libraries were prepared from 1000–4000 ng of RNA using the TruSeq Stranded mRNA kit (Illumina). Library size (about 350 bp) and concentration were assessed with KAPA Library Quantification Kit (Roche). Sequencing was performed on a NextSeq 500/550 (Illumina, 40/40 paired end). Average read numbers were 23.3 million (Huh7-NTCP) and 20.6 million (HepG2.2.15). RNA-seq data were analyzed using CLC Genomics Workbench v25.0.1 (Qiagen, Hilden, Germany). Reads were mapped to the human reference genome (GRCh38) and transcript per million (TPM) values were calculated using default setting. Analyzed data are in the Supplementary Data. Gene sets were analyzed using GSEA Desktop v4.4.0 [31] and Metascape accessed in July 2026 [32]. Broad chromatin accessibility changes identified by bulk ATAC-seq were interpreted together with RNA-seq pathway enrichment analyses to evaluate whether the observed responses reflected coordinated biological processes rather than widespread nonspecific chromatin disruption.

### 4.6. Statistical Analyses

All statistical analyses were performed using R software (v4.3.2, stats package). Values are presented as mean ± standard error of the mean (SE). Statistical significance between two groups was assessed using unpaired or paired two-tailed Student’s *t*-test as appropriate. When sample sizes were limited or data did not satisfy assumptions of normality, non-parametric analysis was performed using the Wilcoxon signed-rank test or Mann–Whitney U test. A *p*-value < 0.05 was considered statistically significant, and multiple comparisons were adjusted using Bonferroni correction where applicable.

## 5. Conclusions

Accessibility-guided targeting of HBV cccDNA provides a complementary framework to conventional sequence-based approaches for identifying candidate CRISPR/Cas9 target sites. In the present study, chromatin accessibility profiling identified reproducible regions of relatively reduced accessibility, and CRISPR/Cas9 perturbation of one representative accessibility-associated region was consistently associated with reductions in HBV DNA-related signals across complementary HBV experimental systems. Although the mechanistic relationship between chromatin accessibility and susceptibility to genome editing remains to be fully elucidated, the present findings support the use of chromatin accessibility profiling as a proof-of-concept framework for identifying candidate cccDNA target regions for future HBV therapeutic studies.

## Data Availability

Raw sequencing data are deposited in the DNA Data Bank of Japan (DDBJ) under accession numbers DRR751184 (scATAC-1), DRR751185 (scATAC-2), and DRR751128-DRR751129 (bulk ATAC-seq).

## Supplementary Materials

The following supporting information can be downloaded at: https://www.mdpi.com/article/10.3390/ijms27156980/s1.

## Abbreviations

The following abbreviations are used in this manuscript:

cccDNA	Covalently closed circular DNA
rOCR	Rarely open chromatin region
ATAC-seq	Assay for transposase-accessible chromatin sequencing
