# Supplementary material for: Chromatin Accessibility-Guided Targeting Identifies Structurally Constrained Regions in HBV cccDNA and Suppresses Viral Replication
Source: Int J Mol Sci. 2026 Aug 3;27(15):6980. doi: 10.3390/ijms27156980 (PMC13466757; doi:10.3390/ijms27156980)
Supplement: Supplementary file 1 [file ijms-27-06980-s001.zip › Supplementary_Files/Supplementary_Figures.pdf]

Supplementary Figures

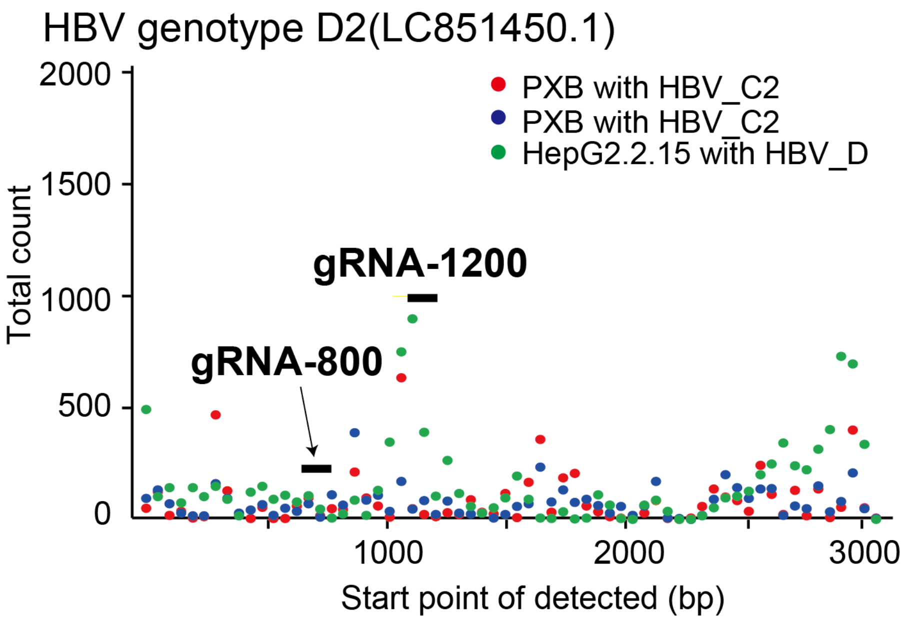

Supplementary Figure S1. Selection of an additional guide RNA target region for comparison with the accessibility-associated 800-bp target. The accessibility profile of the HBV genotype D2 genome (LC851450.1), obtained from bulk ATAC-seq analysis of HepG2.2.15 cells, was used to identify an additional candidate target region. The original guide RNA (gRNA-800) targeted nucleotides 815–834 with the adjacent PAM sequence at nucleotides 835–837, corresponding to the accessibility-associated region identified by single-cell ATAC-seq. An alternative guide RNA (gRNA-1200) was designed to target nucleotides 1188–1207 with the PAM sequence at nucleotides 1208–1210. These guide RNAs were subsequently compared in functional CRISPR/Cas9 experiments to evaluate whether antiviral activity differed between target regions.

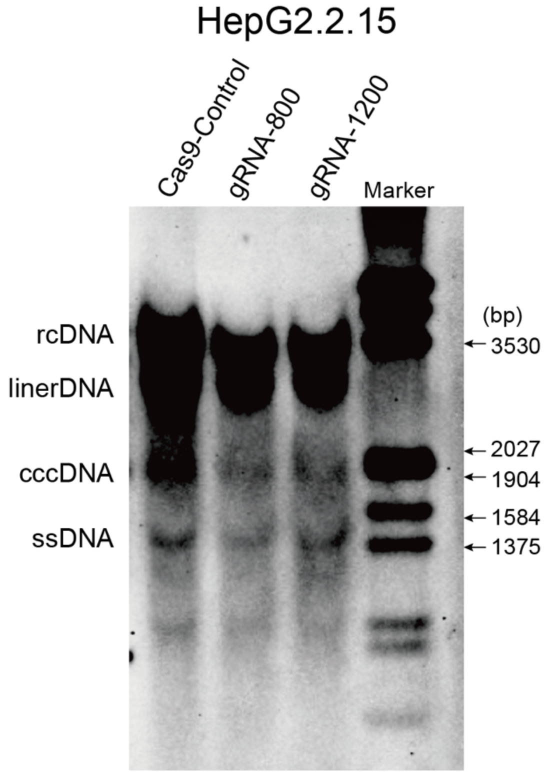

Supplementary Figure S2. Comparison of Southern blot analysis between gRNA-800 and gRNA-1200 targeting different HBV genomic regions. HepG2.2.15 cells were transfected with Cas9 control, gRNA-800, or gRNA-1200. HBV DNA species were analyzed by Southern blotting. The gRNA-800 target corresponds to the accessibility-associated region identified by single-cell ATAC-seq, whereas gRNA-1200 targets an alternative HBV genomic region identified from bulk ATAC-seq analysis of HepG2.2.15 cells. Representative Southern blot images are shown. The gRNA-800 construct showed a greater reduction in cccDNA-associated and other HBV DNA signals than the gRNA-1200 construct. Although this comparison does not establish a causal role for chromatin accessibility, it supports the possibility that antiviral efficacy differs depending on the targeted HBV genomic region.

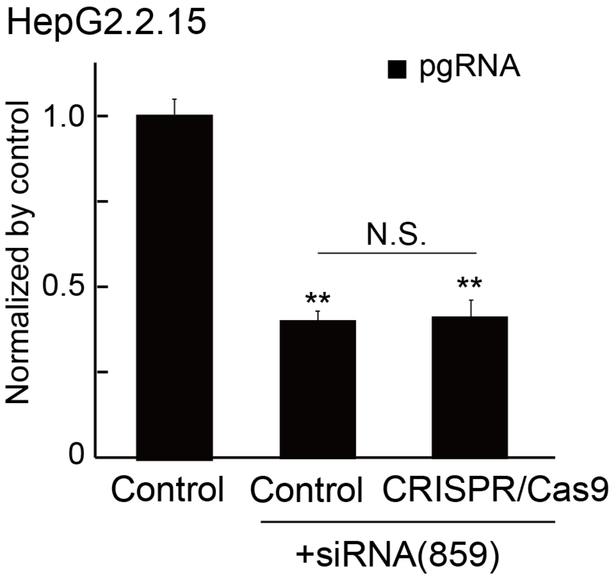

Supplementary Figure S3. Effect of combined CRISPR/Cas9 and siRNA targeting of the same HBV genomic region. An siRNA targeting the HBV 859-bp region, identified in our previous genome-wide siRNA screening, was used in combination with CRISPR/Cas9 targeting the accessibility-associated 800-bp region. HepG2.2.15 cells were treated with control or CRISPR/Cas9 constructs in the presence of the 859-bp-targeting siRNA, and pgRNA expression was quantified by RT-qPCR. Although the siRNA alone significantly reduced pgRNA expression, no additional reduction was observed following combined CRISPR/Cas9 treatment, indicating that simultaneous targeting of the same HBV genomic region by two independent nucleic acid-based approaches did not produce an additive or synergistic antiviral effect. **Data are presented as mean ± SE. $p < 0.01$ versus the control group. N.S. indicates no significant difference between the siRNA-treated control and siRNA-treated CRISPR/Cas9 groups.

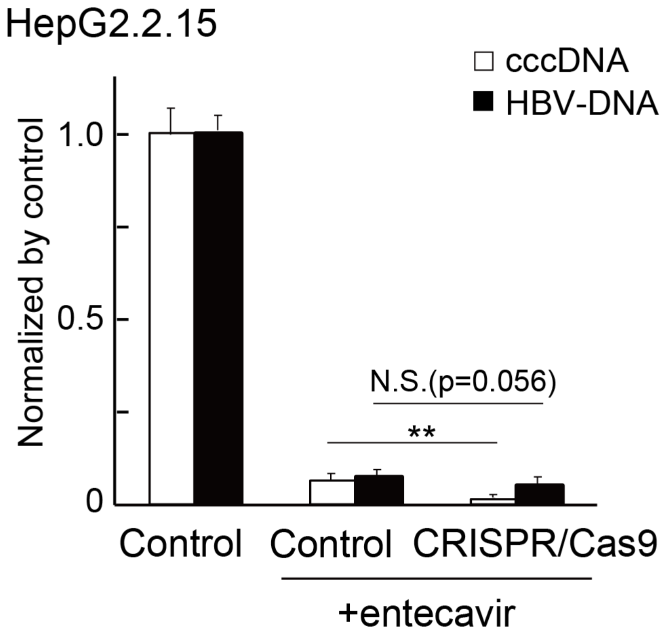

Supplementary Figure S4. Effect of combined CRISPR/Cas9 and entecavir treatment on HBV DNA and cccDNA levels. HepG2.2.15 cells were treated with entecavir alone or in combination with CRISPR/Cas9 targeting the accessibility-associated 800-bp region. Total HBV DNA and cccDNA were quantified by qPCR and normalized to the untreated control. While combined treatment did not further reduce total HBV DNA compared with entecavir alone, a trend toward additional reduction of cccDNA was observed ($p = 0.056$). These findings suggest that CRISPR/Cas9 may provide additional suppression of cccDNA beyond that achieved by entecavir, although this difference did not reach statistical significance under the present experimental conditions. **Data are presented as mean ± SE. $p < 0.01$ versus the entecavir-treated control group for cccDNA. N.S. indicates no significant difference in cccDNA between the entecavir-treated control and the entecavir plus CRISPR/Cas9 groups ($p = 0.056$).

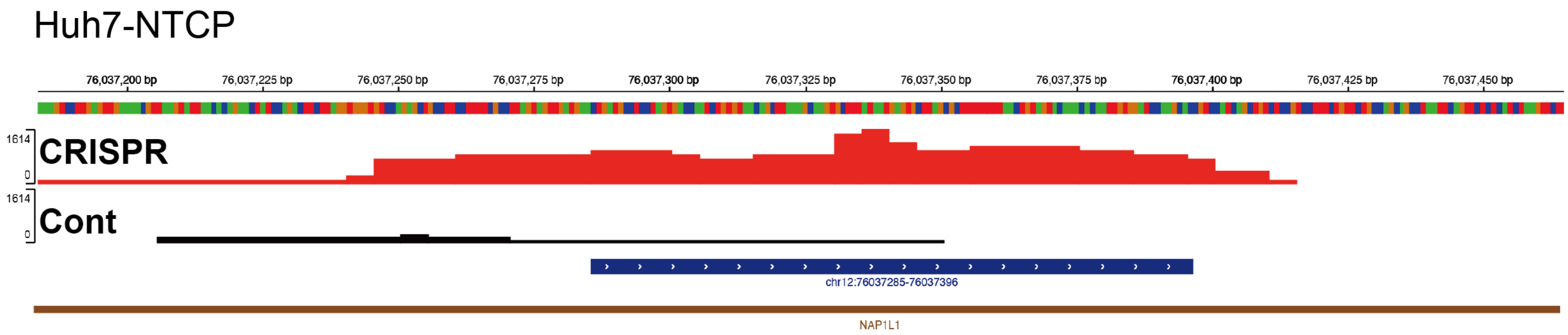

Supplementary Figure S5. Representative ATAC-seq tracks at the NAP1L1 locus. Representative ATAC-seq tracks showing chromatin accessibility at the *NAP1L1* locus in CRISPR/Cas9-treated and control NTCP-Huh7 cells. Increased ATAC-seq signal was observed in CRISPR/Cas9-treated cells compared with control cells at this representative locus. The gene structure of *NAP1L1* is shown below the tracks.

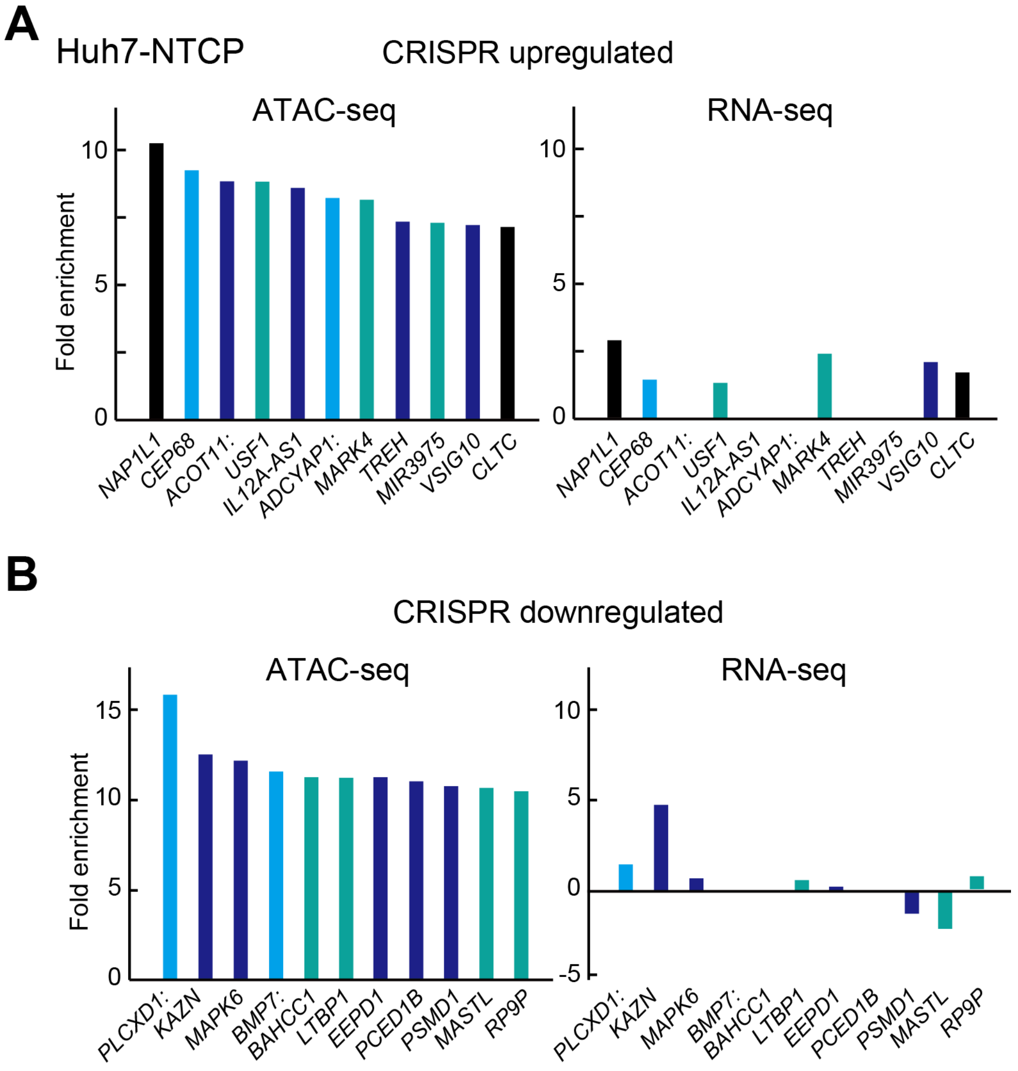

Supplementary Figure S6. Representative genes exhibiting chromatin accessibility changes following CRISPR/Cas9 treatment. **A:** Representative genes showing increased chromatin accessibility detected by ATAC-seq (left) and their corresponding RNA expression changes measured by RNA-seq (right). Bar colors indicate the genomic location of the associated ATAC-seq peaks. **B:** Representative genes showing decreased chromatin accessibility detected by ATAC-seq (left) and their corresponding RNA expression changes measured by RNA-seq (right). Bar colors indicate the genomic location of the associated ATAC-seq peaks.

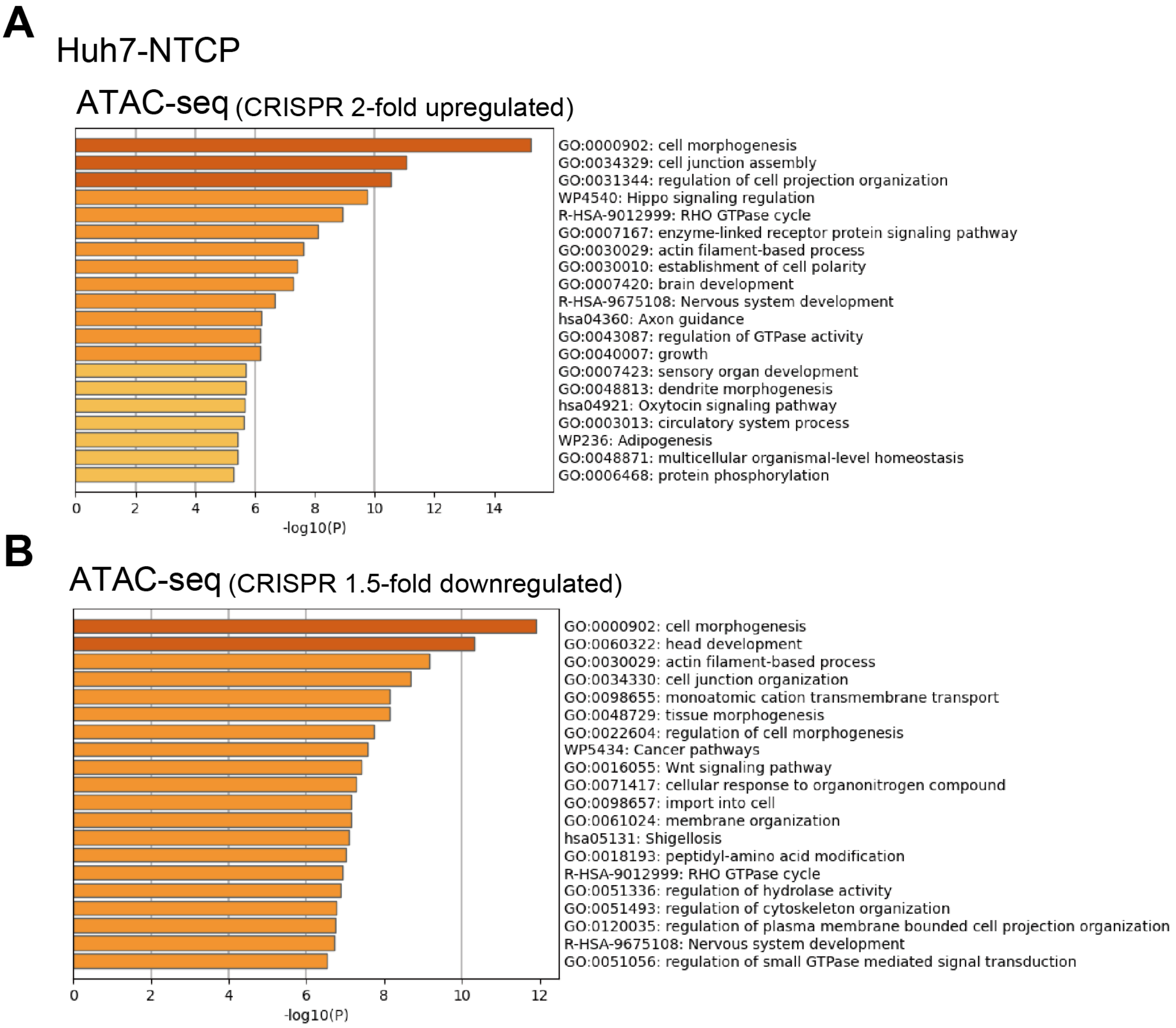

Supplementary Figure S7. Metascape functional enrichment analysis of differentially accessible chromatin regions following CRISPR/Cas9 treatment. **A:** Metascape functional enrichment analysis of loci showing increased chromatin accessibility (>2-fold) identified by ATAC-seq. **B:** Metascape functional enrichment analysis of loci showing decreased chromatin accessibility (>1.5-fold) identified by ATAC-seq. Representative enriched Gene Ontology (GO) terms are ranked according to enrichment significance ($-\log_{10}$ *p* value).

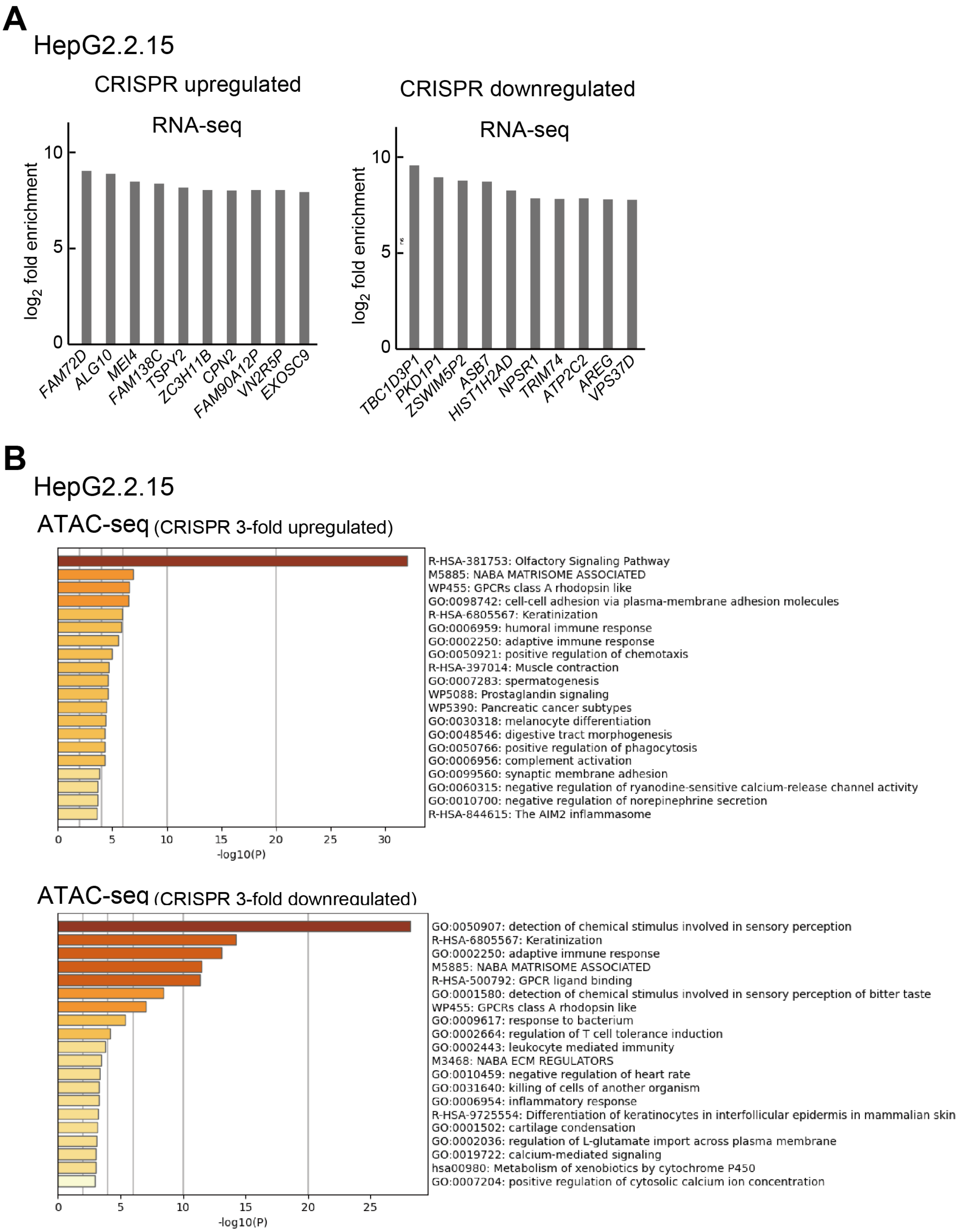
A
HepG2.2.15
CRISPR upregulated
RNA-seq
log2 fold enrichment
0
5
10
FAM72D
ALG10
MEI4
FAM138C
TSPY2
ZC3H11B
CPN2
FAM90A12P
VN2R5P
EXOSC9
CRISPR downregulated
RNA-seq
log2 fold enrichment
0
5
10
TBC1D3P1
PKD1P1
ZSWIM5P2
ASB7
HIST1H2AD
NPSR1
TRIM74
ATP2C2
AREG
VPS37D
B
HepG2.2.15
ATAC-seq (CRISPR 3-fold upregulated)
R-HSA-381753: Olfactory Signaling Pathway
M5885: NABA MATRISOME ASSOCIATED
WP455: GPCRs class A rhodopsin like
GO:0098742: cell-cell adhesion via plasma-membrane adhesion molecules
R-HSA-6805567: Keratinization
GO:0006959: humoral immune response
GO:0002250: adaptive immune response
GO:0050921: positive regulation of chemotaxis
R-HSA-397014: Muscle contraction
GO:0007283: spermatogenesis
WP5088: Prostaglandin signaling
WP5390: Pancreatic cancer subtypes
GO:0030318: melanocyte differentiation
GO:0048546: digestive tract morphogenesis
GO:0050766: positive regulation of phagocytosis
GO:0006956: complement activation
GO:0099560: synaptic membrane adhesion
GO:0060315: negative regulation of ryanodine-sensitive calcium-release channel activity
GO:0010700: negative regulation of norepinephrine secretion
R-HSA-844615: The AIM2 inflammasome
0
5
10
15
20
25
30
-log10(P)
ATAC-seq (CRISPR 3-fold downregulated)
GO:0050907: detection of chemical stimulus involved in sensory perception
R-HSA-6805567: Keratinization
GO:0002250: adaptive immune response
M5885: NABA MATRISOME ASSOCIATED
R-HSA-500792: GPCR ligand binding
GO:0001580: detection of chemical stimulus involved in sensory perception of bitter taste
WP455: GPCRs class A rhodopsin like
GO:0009617: response to bacterium
GO:0002664: regulation of T cell tolerance induction
GO:0002443: leukocyte mediated immunity
M3468: NABA ECM REGULATORS
GO:0010459: negative regulation of heart rate
GO:0031640: killing of cells of another organism
GO:0006954: inflammatory response
R-HSA-9725554: Differentiation of keratinocytes in interfollicular epidermis in mammalian skin
GO:0001502: cartilage condensation
GO:0002036: regulation of L-glutamate import across plasma membrane
GO:0019722: calcium-mediated signaling
hsa00980: Metabolism of xenobiotics by cytochrome P450
GO:0007204: positive regulation of cytosolic calcium ion concentration
0
5
10
15
20
25
-log10(P)

Supplementary Figure S8. Transcriptomic and chromatin accessibility analyses in CRISPR/Cas9-treated HepG2.2.15 cells. **A:** Top genes showing increased (left) or decreased (right) expression in CRISPR/Cas9-treated HepG2.2.15 cells identified by RNA-seq. Genes are ranked according to log_2 fold change. **B:** Metascape functional enrichment analysis of loci showing increased (upper panel; >3-fold) or decreased (lower panel; >3-fold) chromatin accessibility identified by ATAC-seq in CRISPR/Cas9-treated HepG2.2.15 cells. Representative enriched GO terms are ranked according to enrichment significance ($-log_{10}$ p value).
